# Multivariate mixed models accounting for don’t know options in ordinal data

**DOI:** 10.1093/jrsssa/qnaf197

**Published:** 2025-12-26

**Authors:** Ralitza Gueorguieva, Maria Iannario

**Affiliations:** Department of Biostatistics, Yale School of Public Health, 300 George St, New Haven, CT 06511, USA; Department of Political Science, University of Naples Federico II, Via Leopoldo Rodinò 22, Naples 80138, Italy

**Keywords:** cumulative logit model, joint modelling, location scale model, location shift model, random effects, semi-ordinal data

## Abstract

Multivariate ordinal data are common in surveys measuring perceptions or knowledge. Frequently, a ‘don’t know’ option is also available resulting in partially ordinal scales. This complicates modelling: standard ordinal models become inappropriate whereas nominal models are inefficient and difficult to interpret. Simply discarding the ‘don’t know’ responses may lead to bias as responders who choose to provide ratings often differ systematically from those who do not. This manuscript proposes a unified solution that jointly models the selection of ‘don’t know’ options and the ordinal ratings on multiple variables. The proposed multivariate mixed models are flexible, allow for heterogeneity, response styles, and covariate effects both on the ordinal ratings and on choosing the ‘don’t know’ options. Likelihood-based inference and model comparisons are performed. Two case studies: one on financial risk perceptions and one on knowledge about the addictiveness of tobacco products are used for motivation and illustration. A simulation demonstrates that the proposed approach yields unbiased and efficient estimates. The methodology provides interpretable results while capturing the complexity of partially ordinal data. It offers a robust framework for analysing survey responses in social and behavioural research, where ‘don’t know’ options and heterogeneous rating behavior are pervasive.

## 1 Introduction

Ordinal data are widely used in various analytical contexts especially when surveys present several questions with responses with ordered categories. For example, individuals may be asked about their perception of addictiveness of different tobacco products with responses on a five-point scale ranging from ‘very unlikely’ to ‘very likely’ or respondents may be asked about their expectations regarding future inflation rates, with responses ranging from ‘strongly decrease’ to ‘strongly increase’. Cumulative logit regression models are commonly implemented to analyse such data (McCullagh, 1980), often motivated by the existence of continuous latent variables underlying the observed responses. In the examples above, the underlying latent variables are conceptualized as the true perception of an individual about addictiveness of a tobacco product or expectations regarding future inflation but only the categorization into one of several ordinal categories is observed. The classical cumulative-logit models allow for the estimation of the effects of predictors on the location of the latent variable and on the probabilities of response in lower vs. higher categories. For example, in studies on tobacco use, individual characteristics such as current consumption patterns may influence the perceived addictiveness of a given product. Similarly, in the context of economic expectations, past experiences with price changes or broader economic conditions may affect an individual’s forecast of future inflation. However, the cumulative-logit model makes the restrictive assumption that predictors simply shift the response lower or higher and don’t allow for differences in variability of responses or different response styles. Failure to capture this additional subjectivity in the response process, where individuals express perceptions or feelings on certain issues, may lead to bias in estimates and failure to capture meaningful trends (Tutz, 2022). In some cases, respondents choose an option without taking time to give the optimal answer (Krosnick, 1999) or choose an answer without content evaluation (see Van Vaerenbergh & Thomas, 2013, among others). Location scale models (McCullagh, 1980) have been proposed to account for the additional heterogeneity due to the high uncertainty in the responses provided by some individuals vs. others. Location shift models (Tutz & Berger, 2016) have been formulated to account for different responses styles (i.e. the tendency to choose responses towards the middle of the ordinal scale or towards the extremes). Thus, the latter two alternatives, the location-scale and location-shift extensions provide great flexibility in modelling ordinal responses.

However, the inclusion of a ‘don’t know’ (DK) response option frequently compromises the ordinal nature of the data, thereby limiting the direct applicability of standard ordinal modelling approaches. Ignoring the DK responses may lead to biased estimation and incorrect inferences because individuals who reply with DK on some questions may differ from those who choose an ordinal category. In addition, when interviewees answer multiple related survey items and provide a mix of ordinal and DK responses, exploiting the multivariate nature of the data and accounting for within-subject correlations can lead to more accurate and efficient inference. Since the structure of the scales becomes partially ordinal when DK responses are present, mixture models have been proposed (see Gueorguieva et al., 2020 and references therein). These models no longer fit in the general classical framework. They rely on random effects to account for the correlation between repeated measures within individual and to link the ordered responses to the propensity to pick the DK option. However, only mixture cumulative logit or probit models have been considered which do not allow for additional between-subject heterogeneity and different response styles.

With partially ordinal data the same uncertainty in the response process due to between-subject differences may be observed. For example, some individuals may have tendency to provide answers in middle categories while other tend to provide more extreme answers. The large literature on mixture models for the analysis of ordinal data with heterogeneity (D’Elia & Piccolo, 2005; Piccolo, 2003; Piccolo & Simone, 2019) and different response styles (Tutz, 2022) encounters limitations in the possible extensions (see for the latter Iannario et al., 2020; Manisera & Zuccolotto, 2014). The limitations focus on the one-dimensionality of the approach as it is unable to deal with multiple ordinal response variables at the same time.

This article presents a new methodological idea that considers DK as valid responses and models them together with the ordinal responses. This approach allows estimation of the effects of covariates on DK responses and on ordinal responses, and is also capable of capturing differences in response styles as well as handling excessive variability in the data. The proposal generalizes the cumulative logit, the location scale and the location shift models with multiple response variables. Therefore, it can be seen as an extension of the work of Hedeker et al. (2009) and of the Partial Credit Model Response Style (Tutz et al., 2018). The contribution of Hedeker et al. (2009) constitutes a mixed-effects logistic regression model for the analysis of ordinal longitudinal outcomes in which the standard mixed logistic model is extended by adding a subject-level random effect to the within subject variance specification. This allows individuals to vary in the mean, or position, and the variance, or square of the scale, of their responses. The limitation in this case, overcome by our proposed model, concerns the presence of the DK option that we specifically model. Similarly, this is the extension we make with respect to the work of Tutz et al. (2018) who in the context of Item Response Theory explicitly introduce person-specific response style parameters modelling a tendency to extreme or middle categories.

The multivariate generalization we propose allows multiple items to be taken into account simultaneously and uses the structure of mixed-effects models as proposed in other approaches such as the generalized linear latent variable models (Skrondal & Rabe-Hesketh, 2004), also denoted as Hierarchical Generalized Linear Models (Raudenbush & Bryk, 2002), and, more precisely, as two-dimensional IRT models (Reckase, 2009) with related latent traits that extend the generalized linear Item Response Theory models to the bivariate case (Mellenbergh, 1994).

Our methodological proposal takes into account three aspects: (i) considers the association among different responses in questionnaires consisting of multiple questions on an ordinal scale; (ii) assesses the possible presence of variability and of response styles in the selection process for these items; (iii) deals with the possible selection of the DK option expressed by respondents that may influence the measurement of the latent variable driving the selection process.

The proposed models, model fitting and inference procedures are in Section 2. Sections 3 and 4 present the results of the analysis of two case studies: one evaluating financial knowledge and another evaluating the perception about the addictiveness of tobacco products. Section 5 presents the methodology and results of a simulation study conducted to evaluate the performance of the proposed models in terms of parameter estimation and statistical inference. Our concluding remarks are in Section 6.

## 2 Methods

We consider a scenario where each individual *i* in a sample of *N* individuals is asked a series of related questions (j=1,…J) and provides responses $y_{ij}$ to those questions on an ordinal scale (coded without loss of generality as 1,2,…C) with the additional option to respond DK to each question (coded as C+1). Typically, higher responses (up to category C) correspond to agreement with the statement in each question, and lower scores correspond to disagreement, whereas scores in the middle reflect neither agreement nor disagreement (although this is not a requirement). The inclusion of a DK option disrupts the ordinal structure of the data, while treating all responses as nominal proves to be inefficient and fails to yield meaningful estimates. To address this challenge, we propose the development of mixture models that incorporate logistic components to handle DK responses and ordinal components to effectively analyse the remaining data, preserving the integrity and efficiency of the analysis. In the model formulations presented below, the logistic components remain consistent across all variations. However, we explore three distinct approaches for the ordinal components, each tailored to capture different aspects of the data’s structure and characteristics: cumulative logit, location-scale cumulative logit, and location-shift cumulative logit models. Here, the term *location* refers to the placement of thresholds along the latent variable, indicating the points at which respondents transition between adjacent categories on the ordinal response scale.

Let $y_{ij}^{c}=I\{y_{ij}\neq C+1\}$ and $\pi_{ij}=P\{y_{ij}\neq C+1\}$, and we assume that $y_{ij}^{c}$ has Bernoulli distribution with probability $\pi_{ij}$ (interpreted as the propensity of individual *i* to provide a rating to question j). Let also $\pi_{ijk}=P\{y_{ij}\leq k|y_{ij}\neq C+1\}$ denote the cumulative conditional probability for response variable *j* up to category *k* if an ordinal rating is provided, k=1,…C−1. Following standard notation β is used to denote a vector of regression coefficients for fixed effects, x is a vector of fixed predictors, *λ* is a factor loading for the random effect, *b*—random intercept and *τ*—threshold parameter. Subscript *i* denotes individual, *j* denotes dependent variable, and *k* denotes response category of the ordinal responses. Individuals are assumed to be independent of each other, and repeated responses within individual are assumed to be conditionally independent given the random effects. **D** denotes a correlation matrix (that is, with unit diagonal elements and off-diagonal elements between −1 and 1).

Formulation 1 (**LCL**  =  **Logit/Cumulative Logit Model**):

$$\begin{array}{rl} \ln\;\frac{\pi_{ij}}{1-\pi_{ij}} & =x_{0ij}^{T}\beta_{0j}+\lambda_{0}b_{i0}\\ \ln\;\frac{\pi_{ijk}}{1-\pi_{ijk}} & =\tau_{\;jk}+x_{ij}^{T}\beta_{\;j}+\lambda_{\;j}b_{i1}\\ (b_{i0},b_{i1}{)}^{T} & ∼i.i.d.N(0,D) \end{array}$$

Formulation 1 corresponds to the most parsimonious joint model and allows for between-subject variability in location, but it does not allow for between-subject variability in scale across the different response categories. Adding a scale component leads to Formulation 2. In this formulation, the logarithm of the variance ${\sigma_{j}}^{2}$ of the ordinal components is also related to covariates, $z_{ij}$ is a covariate vector for variable *j* on individual *i*, and $\gamma_{j}$ is a vector of unknown coefficients for variable *j*. The covariates contained in the predictor vector $z_{ij}$ may either constitute a subset of $x_{ij}$ or represent variables specific to the response scale. Note that in this formulation the presence of covariates for the variances ${\sigma_{j}}^{2}$ is required for model identifiability.

Formulation 2 (**LLCM**  =  **Logit/Location sCale Model**):

$$\begin{array}{rl} \ln\;\frac{\pi_{ij}}{1-\pi_{ij}} & =x_{0ij}^{T}\beta_{0j}+\lambda_{0}b_{i0}\\ \ln\;\frac{\pi_{ijk}}{1-\pi_{ijk}} & =\frac{\tau_{\;jk}+x_{ij}^{T}\beta_{\;j}+\lambda_{\;j}b_{i1}}{\sigma_{j}}\\ {\sigma_{j}}^{2} & =\exp\;(z_{ij}^{T}\gamma_{\;j})\\ (b_{i0},b_{i1}{)}^{T} & ∼i.i.d.N(0,D). \end{array}$$

Another way to extend Formulation 1 is to allow the thresholds for the ordinal response variables to vary depending on covariates. This could reflect different response styles (e.g. answers towards the middle of the ordinal scale, or answers in extreme categories) of different individuals. In this formulation, there is a shift in the thresholds delineating the responses categories for the ordinal dependent variables. This shift is assumed to be influenced by covariates, a requirement necessary to ensure parameter identifiability. We follow the recommended specification by Tutz et al. (2018). Herein $z_{ij}$ refers to a vector of covariates for the response category shift for response variable *j* and the $\gamma_{\;j}$ is the corresponding coefficient vector.

Formulation 3 (**LLHM**  =  **Logit/Location sHift Model**)

$$\begin{array}{rl} \ln\;\frac{\pi_{ij}}{1-\pi_{ij}} & =x_{0ij}^{T}\beta_{0j}+\lambda_{0}b_{i0}\\ \ln\;\frac{\pi_{ijk}}{1-\pi_{ijk}} & =\tau_{\;jk}^{*}+x_{ij}^{T}\beta_{\;j}+\lambda_{\;j}b_{i1}\\ \tau_{\;jk}^{*} & =\tau_{\;jk}+(C/2-k)z_{ij}^{T}\gamma_{\;j}\\ (b_{i0},b_{i1}{)}^{T} & ∼i.i.d.N(0,D). \end{array}$$

In our model formulations, identifiability constraints are needed in the scale equation in Formulation 2 and in the shift equation in Formulation 3 where we set intercepts equal to zero. Of note, the parameters in the two components of our models (the random intercept logistic model for the binary DK responses and the random intercept models for the ordinal responses) are identifiable provided that standard guidelines are observed (Agresti, 2013; Rabe-Hesketh & Skrondal, 2001) and hence the joint models are also identifiable provided that there is co-occurrence of DK and ordinal responses for some individuals. If the sample is completely separable into a subset with only DK responses and a subset with only ordinal responses, then we will not be able to identify the correlation parameter linking the two models. Additionally, the assumption of common random intercepts across the DK and ordinal components further supports identifiability by constraining the latent structure and leveraging the dependency across items. This specification ensures that subject-specific tendencies to respond DK or to choose higher ordinal categories are captured consistently across questions, thereby avoiding underidentification due to overparametrization.

While the fixed intercepts/thresholds and regression parameters capture common effects across individuals, the random shared intercepts account for individual variations, reflecting a higher or lower tendency to provide DK responses and a greater or lesser inclination to give higher ratings when ratings are provided regardless of the question being asked. Thus, we assume that individual deviations from the mean responses remain consistent across variables, though the magnitude of these deviations can differ for the ordinal responses (allowing for distinct factor loadings, denoted by *λ*). This flexible framework accommodates varying correlations between responses within individuals, enabling a nuanced representation of individual-level dependencies across different response types.

The likelihood function in all three model formulations is obtained by integrating over the random effects. We present the likelihood for the first formulation. The likelihoods for the other two formulations are obtained similarly with the addition of the scale and shift parameters for Formulations 2 and 3, respectively, and changing the linear predictors accordingly. To specify the likelihood, let $f(b_{i0},b_{i1};D)$ be the multivariate normal density of the random effects, and let $y_{ijk}=I\{y_{ij}=k\}$, k=1,2,…C. Then the marginal likelihood is written as


$$\prod_{i=1}^{N}\int_{R^{2}}\prod_{\;j=1}^{J}\left(g(y_{ij}^{c}|b_{i0};\beta_{0})\prod_{k=1}^{C}g(y_{ijk}|b_{i1};\beta_{j})\right)f(b_{i0},b_{i1};D)db_{i0}db_{i1},$$

where


$$\begin{array}{rl} g(y_{ij}^{c}|b_{i0};\beta_{0j}) & =\frac{\exp\;\left((x_{0ij}^{T}\beta_{0j}+\lambda_{0}b_{i0})y_{ij}^{c}\right)}{1+\exp\;(x_{0ij}^{T}\beta_{0j}+\lambda_{0}b_{i0})},\\ g(y_{ij1}|b_{i1};\beta_{j}) & ={\left(\frac{\exp\;(\tau_{\;j1}+x_{ij}^{T}\beta_{j}+\lambda_{\;j}b_{i1})}{1+\exp\;(\tau_{\;j1}+x_{ij}^{T}\beta_{j}+\lambda_{\;j}b_{i1})}\right)}^{y_{ij1}},\\ g(y_{ijk}|b_{i1};\beta_{j}) & =(\frac{\exp\;(\tau_{\;jk}+x_{ij}^{T}\beta_{j}+\lambda_{\;j}b_{i1})}{1+\exp\;(\tau_{\;jk}+x_{ij}^{T}\beta_{j}+\lambda_{\;j}b_{i1})}\\ & \;{-\frac{\exp\;(\tau_{\;j,k-1}+x_{ij}^{T}\beta_{j}+\lambda_{\;j}b_{i1})}{1+\exp\;(\tau_{\;j,k-1}+x_{ij}^{T}\beta_{j}+\lambda_{\;j}b_{i1})})}^{y_{ijk}}, \end{array}$$

where k=2,…C−1 and


$$g(y_{ijC}|b_{i1};\beta_{j})={\left(\frac{1}{1+\exp\;(\tau_{\;j,C-1}+x_{ij}^{T}\beta_{j}+\lambda_{\;j}b_{i1})}\right)}^{y_{ijC}}.$$

The multidimensional integral above is intractable, and some numerical, stochastic, or analytical approximation must be used. We choose to use numerical maximization in the form of adaptive Gaussian quadrature, which is readily available in statistical software.

We use ψ to denote the vector of all parameters, i.e. all regression (*β*), threshold (*τ*), factor loading (*λ*), and correlation parameters (off diagonal elements of D), and y to denote a column vector of all observed responses. Also, $y_{i}$ is a column vector of all observed responses on individual *i* and $b_{i}$ is the vector of the random effects for subject *i*. The marginal log-likelihood is then written as


$$\ln\;L(y;\psi )=\sum_{i=1}^{N}\ln\;L_{i}(y_{i};\psi ),$$

where


$$\begin{array}{rl} L_{i}(y_{i},\psi ) & =\int_{R^{2}}\prod_{\;j=1}^{J}\left(g(y_{ij}^{c}|b_{i0};\beta_{0j})\prod_{k=1}^{C}g(y_{ijk}|b_{i1};\beta_{j})\right)\\ & \;\;\frac{1}{|2\pi D{|}^{\frac{1}{2}}}\exp\;\left(-\frac{1}{2}b_{i}^{T}D^{-1}b_{i}\right)db_{i}, \end{array}$$

$L_{i}(y_{i};\psi )$ is the conditional likelihood function for individual *i* and β is a vector of all the intercept and fixed regression parameters. In Formulations 2 and 3, the conditional likelihoods for the ordered categories also depend on the additional scale and shift parameters.

Individual likelihoods $L_{i}(y_{i},\psi )$ are approximated using adaptive Gauss–Hermite quadrature (Liu & Pierce, 1994) such that at each step the integrals are approximated by a weighted sum of the integrands at quadrature points around the mode of the posterior distribution of the random effect. We denote the approximation by $L_{i}^{GQ}$. To obtain maximum likelihood estimates, we follow the steps outlines in Table 1.

**Table 1.** The general form of the iterative maximum likelihood estimation procedure

[1]	We choose a starting estimate for ψ (${\hat{\psi }}^{\left(0\right)}$) and set a counter l=0.
[2]	Increase *l* by 1. Approximate each of the integrals $L_{i}(y_{i};{\hat{\psi }}^{\left(l-1\right)})$ by $L_{i}^{GQ}(y_{i};{\hat{\psi }}^{\left(l-1\right)}).$
[3]	Update the estimate of ψ to ${\hat{\psi }}^{\left(l\right)}$ using a numerical maximization routine.
[4]	Iterate between steps 2 and 3 until convergence.

Initial estimates are chosen to be neutral values, for example, zeros for the regression parameter estimates, ones for variance components, consecutive integer values for the threshold parameters. To increase the likelihood of convergence to the global maximum rather than a local one, multiple sets of starting values are employed. Convergence is achieved when the relative difference between likelihood function approximations in subsequent iterations becomes smaller than a prespecified small value. Upon convergence estimates of the standard errors of the parameter estimates are based on the observed information matrix after the algorithm converges. Numerical derivatives are necessary to approximate the observed information matrix. The general theory of maximum likelihood applies and hence the estimates are expected to be unbiased and asymptotically efficient. Full maximum likelihood estimation, hypothesis testing, and confidence interval estimation are performed using SAS PROC NLMIXED. Model selection is based on the Bayesian Information Criterion (BIC) (Schwarz, 1978) and the model with the lowest BIC is selected. Likelihood-ratio tests can be performed for nested models for which the parameters are not on the boundary of the parameter space.

To evaluate how well the fitted data match the observed data for a particular model we also compute the proportion of correctly classified responses (CCR) across all ordinal responses and the DK options. Furthermore, we adapt some of the normalized indices from the literature for assessing the predictive power and the fit of a model with the additional presence of DK answers. For example, we consider the index:


$$Pseudo\;R^{2}=1-\frac{\ln\;L(y;\hat{\psi })}{\ln\;L_{0}(y;{\hat{\psi }}_{0})}$$

for assessing the fit of a model of interest with likelihood $L(y;\hat{\psi })$ against the independence model with likelihood $L_{0}(y;{\hat{\psi }}_{0})$ characterized by the absence of random effects and fixed predictors, i.e. a model that reduces to separate models for the DK options and the ordinal responses with just thresholds/intercepts. Since there are no covariates in model $L_{0}$, the model is an unconditional ordinal model with ordinality preserved due to the structure of the cumulative link. The pseudo $R^{2}$ is between 0 and 1 with higher values indicating a better predictive power than the independence model (although for non-normal data the index may remain substantially below 1).

As a measure of relative model fit, we also consider the ratio of geometric means of the contributions to the maximized likelihoods of two nested models $M_{R}$ and $M_{F}$ (*R* indicating reduced and *F* indicating full model) proposed by Agresti and Caffo (2002):


$${\hat{\rho }}_{RF}=\exp\;\left\{\frac{1}{N}[\ln\;L_{R}(y;{\hat{\psi }}_{R})-\ln\;L_{F}(y;{\hat{\psi }}_{F})]\right\}$$

which equals to exp(−1/2N) times the usual likelihood-ratio test statistic for comparing the two models. This scaled version of the likelihood-ratio test statistic is easier to interpret and compare across different pairs of models because it does not directly depend on the sample size.

## 3 Fintech knowledge case study

### 3.1 Data

The data consist of 625 Italian respondents to the survey on ‘Knowledge and Use of Fintech Products’ that was conducted as part of the European project CA19130 Fintech and Artificial Intelligence in Finance.^1^

The online self-administered questionnaire asked participants to gauge their knowledge level and perceived relevance of several financial tools, in particular ETF (investment fund) on a six-point ordinal response scale. There were 12 questions in the survey from which we choose the three items with substantial proportion of DK responses. The questions were: ‘What information would you like to have before making decisions?’ (0 = ‘not important’; 5 = ‘extremely important’). Among the items designed to evaluate the understanding of financial instruments, the following three items were selected:

1. Information on *risk* class
2. Past performance *benchmarks*
3. Full description of included *assets*

 Table 2 presents descriptive statistics of variables describing the sample. The median age of the respondents was 23 years old with slightly more females than males (55.4% females and 44.6% males). The income variable was obtained from the responses to the question ‘Household income sufficient to see the family through to the end of the month’ on an ordinal scale from 1 (with great difficulty) to 10 (very easily). The mode of the income distribution was 7 with 63.5% of the sample with a rating above 6. More than half of the respondents lived in Southern Italy, followed by those coming from the North (39.2%); the rest were either from the Center or did not live in Italy at the time of the interview (labelled as ‘Other’). Seven respondents (equivalent to 1.1%) declared not having at least a high school degree. About one-half had a high school diploma whereas 34.4% had a bachelor’s degree. The remainder had master’s degree or higher degree. Focusing on University degree type 16.3% of respondents stated that they came from a course of study in Economics.

**Table 2.** Descriptive statistics of the Fintech knowledge data

		*n*	%
Gender			
	Female	346	55.4
	Male	279	44.6
Area			
	North	245	39.2
	Center	52	8.3
	South/Islands	317	50.7
	Other	11	1.8
Education			
	Primary school	7	1.1
	Diploma	306	49.0
	Bachelor	215	34.4
	Master	76	12.2
	Postgraduate	21	3.4
University degree			
	Economics	102	16.3
	Other	523	83.7
Family component			
	Alone	15	2.4
	2	60	9.6
	3	136	21.8
	4 and more	414	66.2
Income			
	1 - with great difficulty	20	3.2
	2	12	1.9
	3	27	4.3
	4	33	5.3
	5	48	7.8
	6	88	14.2
	7	117	18.7
	8	110	17.6
	9	68	10.9
	10 - very easily	102	16.3
		Mean	SD
Age		24.5	7.3

Figure 1 shows the marginal distribution of the three dependent variables. Overall, DK response proportions varied from about 10% for risk to 19% for benchmark. The responses for risk skewed more towards the upper end of the scale, whereas the distribution for benchmark was close to uniform across the response categories. The modes of the values for both risk and asset were at the upper end of the ordinal scale. About 35% of individuals graded information on risk as extremely important, whereas about 23% graded information on assets as extremely important.

**Figure 1.**
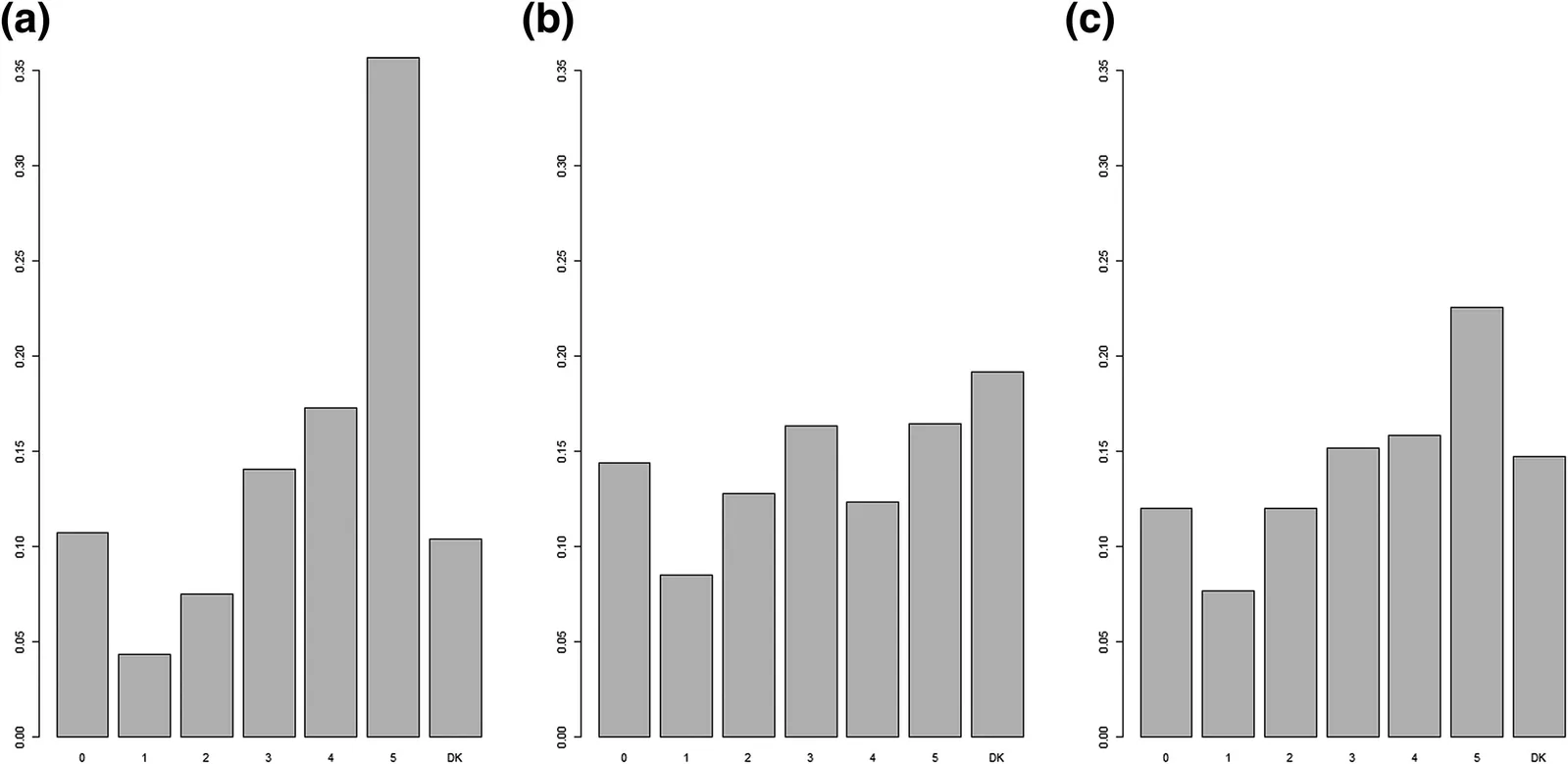
Frequency distribution of the *risk* (a), *benchmarks* (b), *assets* (c) variables in the Fintech knowledge case study.

### 3.2 Results

To determine which fixed predictors to include, we began with exploratory analyses by fitting separate models for each component: cumulative logit, location-scale, and location-shift models for the ordinal responses, and distinct logistic models for the choice between providing a rating versus selecting the DK category. This initial exploration was conducted for computational efficiency, as performing variable selection within the full model could be significantly more demanding. The separate analyses revealed that family size and enrolment in an economics degree program (compared to non-economics programs) were associated with the ordinal ratings. Additionally, age was found to influence the likelihood of selecting more extreme versus central response categories. Lastly, we observed that gender was significantly associated with the likelihood of selecting the DK option, with remarkably consistent regression estimates across the different variables. This observation, coupled with the consistent rates of selecting the DK option across different questions, suggests that the likelihood of choosing the DK response is more of a subject-specific trait than a question-dependent phenomenon. Hence, we assumed the same fixed intercepts and slope effects across questions which is equivalent to treating the number of DK answers per individual as arising from a binomial distribution with the same probability of ‘success’ within individual. The correlation between DK responses from the same individual arises from the individual’s propensity to pick the DK option.

In our multivariate analyses, we applied all three model formulations to the risk, asset, and benchmark responses in the case study using SAS PROC NLMIXED. We employed adaptive Gaussian quadrature with 10 quadrature points per dimension to ensure accurate estimation. In our analyses, we included gender as a regression predictor for DK responses, while economy and family components were used as predictors of location across all three model formulations. Additionally, age was incorporated as a predictor of the shift and scale components in Formulations 2 and 3.

Table 3 provides a comparison of the BICs of the three model formulations with and without correlated random effects. Note that assuming uncorrelated random effects is equivalent to fitting the DK responses separately from the ordered responses. The model fit indices suggest that the location shift model with don’t know (LLHM, Formulation 3) fits the data the best (BIC=5350.4 which is the smallest BIC of the considered models).

**Table 3.** Comparison of joint models fit to the Fintech knowledge data

		BIC
Correlated random effects		
	Cumulative Logit Model with Don’t Know (LCL)	5352.0
	Location Scale Model with Don’t Know (LLCM)	5356.6
	Location Shift Model with Don’t Know (LLHM)	5350.4
Uncorrelated random effects		
	Cumulative Logit Model and Don’t Know	5400.6
	Location Scale Model and Don’t Know	5432.6
	Location Shift Model and Don’t Know	5391.7

Table 4 shows the estimated parameters with standard errors in the best fitting model (i.e. the LLHM model, Formulation 3, in the last column) and in the corresponding location-shift model that ignores the DK option. The models reveal that having a degree in economics is associated with higher ordinal responses, suggesting that individuals with an economics background are more inclined to seek information, as they possess the tools and skills to conduct evaluations independently. Conversely, coming from a larger family is linked to lower ordinal responses, indicating a potential influence of familial dynamics on decision-making and response patterns. Females were more likely to provide a DK response consistent with literature that generally places women at a higher propensity towards this type of response when faced with economic issues (Chen & Garand, 2018). Focusing on response style, older individuals were more likely to provide responses towards the extremes of the ordinal scale compared to younger individuals. This may reflect a growing confidence in decision-making as individuals age, possibly due to accumulated experience and knowledge.

**Table 4.** Results from the Fintech knowledge case study

Component	Parameter	Joint Location Shift	Joint Location Shift and
		Ignoring Don’t Know	Don’t Know (LLHM model)
		Est (SE)	Est (SE)
Rating vs. Don’t know			
	Intercept ($\beta_{00}$)	–	5.11 (0.50)
	Gender ($\beta_{01}$)	–	− 0.82 (0.43)
	Random intercept loading ($\lambda_{0}$)	–	4.10 (0.40)
	Correlation of random intercepts (*ρ*)	–	0.67 (0.06)
Risk			
	Threshold 1 ($\tau_{11}$)	− 8.69 (1.67)	− 9.63 (1.73)
	Threshold 2 ($\tau_{12}$)	− 7.88 (1.54)	− 8.69 (1.59)
	Threshold 3 ($\tau_{13}$)	− 6.84 (1.43)	− 7.55 (1.47)
	Threshold 4 ($\tau_{14}$)	− 5.29 (1.32)	− 5.89 (1.35)
	Threshold 5 ($\tau_{15}$)	−3.91 (1.27)	− 4.42 (1.29)
	Economy ($\beta_{11}$)	− 1.62 (0.70)	− 1.75 (0.70)
	Family components ($\beta_{12}$)	1.07 (0.36)	1.06 (0.36)
	Age on shift ($\gamma_{1}$)	− 0.06 (0.02)	− 0.06 (0.02)
	Random intercept loading ($\lambda_{1}$)	5.47 (0.63)	6.04 (0.70)
Benchmark			
	Threshold 1 ($\tau_{21}$)	− 3.31 (0.88)	− 3.73 (0.89)
	Threshold 2 ($\tau_{22}$)	− 2.71 (0.85)	− 3.09 (0.85)
	Threshold 3 ($\tau_{23}$)	− 2.04 (0.83)	− 2.39 (0.83)
	Threshold 4 ($\tau_{24}$)	− 1.24 (0.82)	− 1.57 (0.82)
	Threshold 5 ($\tau_{25}$)	− 0.64 (0.83)	− 0.97 (0.82)
	Economy ($\beta_{21}$)	− 1.28 (0.45)	− 1.30 (0.44)
	Family components ($\beta_{22}$)	0.48 (0.23)	0.46 (0.22)
	Age on shift ($\gamma_{2}$)	− 0.03 (0.01)	− 0.03 (0.01)
	Random intercept loading ($\lambda_{2}$)	3.36 (0.26)	3.65 (0.29)
Asset			
	Threshold 1 ($\tau_{31}$)	− 7.48 (1.67)	− 7.62 (1.53)
	Threshold 2 ($\tau_{32}$)	− 5.80 (1.49)	− 6.03 (1.37)
	Threshold 3 ($\tau_{33}$)	− 4.16 (1.37)	− 4.50 (1.27)
	Threshold 4 ($\tau_{34}$)	− 2.58 (1.30)	− 3.04 (1.21)
	Threshold 5 ($\tau_{35}$)	− 1.01 (1.29)	− 1.60 (1.20)
	Economy ($\beta_{31}$)	− 1.83 (0.73)	− 1.79 (0.66)
	Family components ($\beta_{32}$)	0.79 (0.37)	0.73 (0.33)
	Age on shift ($\gamma_{3}$)	− 0.03 (0.02)	− 0.03 (0.02)
	Random intercept loading ($\lambda_{3}$)	5.72 (0.72)	5.79 (0.65)

However, even after accounting for differences in university degree, family composition, gender and age, there is still high heterogeneity between individuals in their responses as indicated by the large values of the factor loadings for the random intercepts. The positive signs of the factor loadings for the random intercepts in the ordinal responses suggest a tendency for individuals who provide higher ratings on one question to also give higher ratings on other questions, and similarly, lower ratings tend to be consistent across responses. Furthermore, there is substantial correlation between providing a rating on a question and how high the rating is on other questions, as indicated by the high and significant correlation of the two random intercepts (0.67). In other words, individuals who tend to select the DK response on one question are also more likely to deem the corresponding information as unimportant on another. This pattern may indicate a more indifferent or disengaged approach to financial decision-making among those who frequently rely on the DK response option.

Neglecting the DK options does not appear to significantly alter the estimates of fixed effects when compared to the full multivariate model that includes DK responses. However, the threshold and variance estimates show some attenuation when DK responses are ignored. This potential bias is further examined and validated through the simulation study presented in the next section of the manuscript.

Assessing model fit, Figure 2 shows the correspondence between predicted and observed values for all three (marginal) dependent variables from the model including the DK options. For the purpose of this evaluation, individuals’ predicted ordinal responses for each variable are the categories with the highest probabilities calculated from the model. There appears to be good agreement of the predictions with the observed data from the location-shift model incorporating the DK option. The percentage of correct predictions is 80% for risk, 56% for benchmark and 77% for asset which amounts to 71% correct prediction overall. This is very similar to the prediction from the cumulative-logit model that ignores response style (78% for risk, 57% for benchmark, and 77% for asset which amounts to 71% correct prediction overall). However, the likelihood-ratio test comparing the best-fitting location-shift model with DK (LLHM, Formulation 3) to the cumulative-logit model with DK (LCL, Formulation 1) indicates statistically significantly better fit of the former which takes response style into account (likelihood-ratio test statistic of 20.8 at 3 degrees of freedom, corresponding to a *p*-value of 0.0001). Thus information about response style leads to better fit and understanding of the effects of age but does not translate into substantially better overall prediction. Of note, the predictions for risk and asset are significantly better than for benchmark, reflective of the more information-rich distributions for risk and asset observed in the raw data. Furthermore, there is more uncertainty in responding to the benchmark question compared to the other two questions.

**Figure 2.**
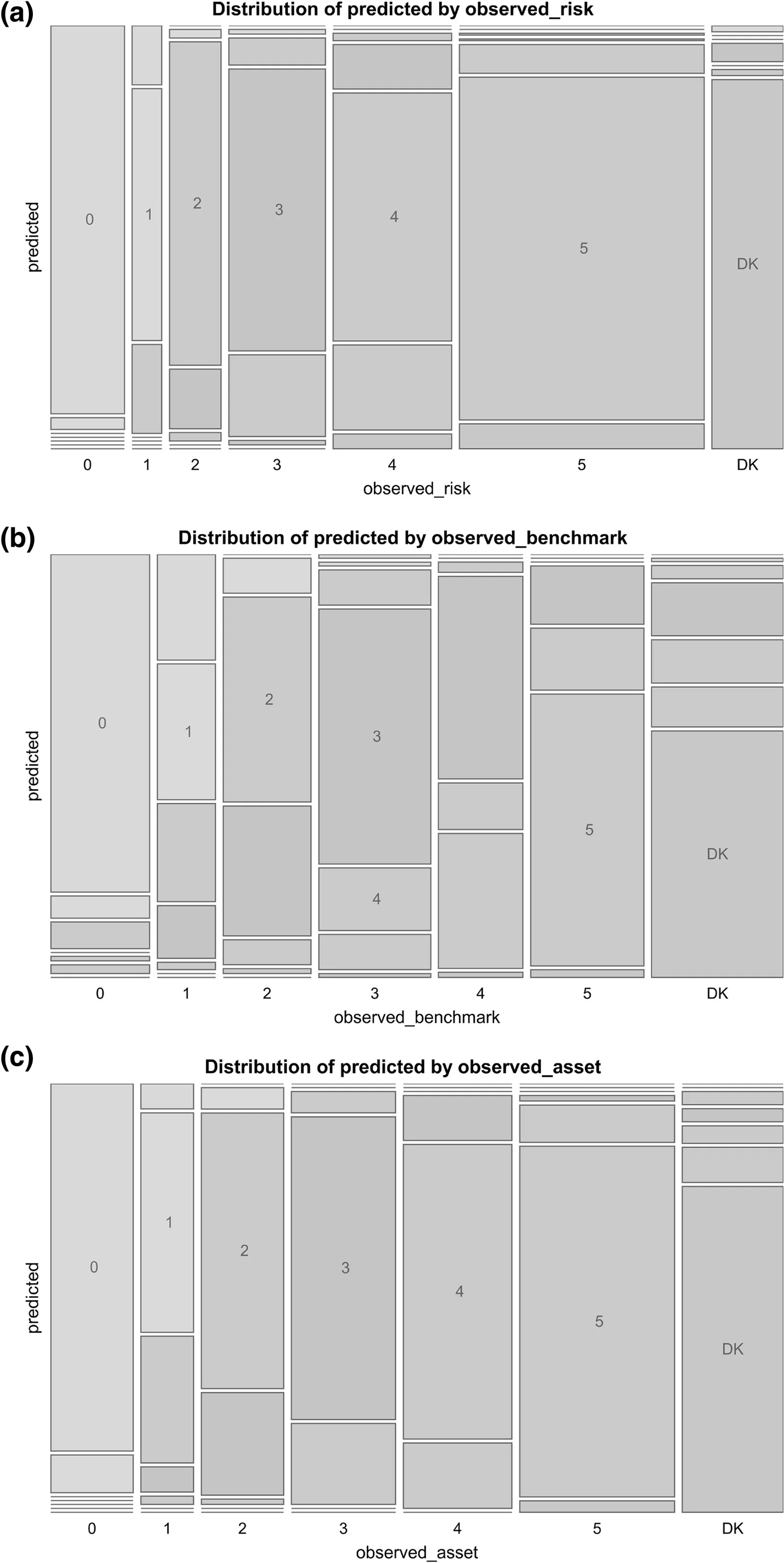
Mosaic plot of observed vs. predicted variables in the logit location shift model fitted to the Fintech knowledge data. (a) Risk variable. (b) Benchmark variable. (c) Asset variable.

The pseudo $R^{2}$ index is 0.24 when comparing the fit of the LLHM model to the fit of a model (herein referred to as the independence model) consisting of three independent sub-models for the ordinal measures of risk, benchmark and asset, and a logistic sub-model for DK responses without covariates and random effects. It is difficult to interpret this value since the data are not normally distributed. The geometric mean index of relative model fit, however, provides a more meaningful comparison between these two models. It is calculated to be ${\hat{\rho }}_{\mathrm{RF}}=0.27$ which indicates that the independence model provides significantly worse fit than the LLHM model as the value is substantially lower than 1. In contrast, the LLHM model and the LCL model have similar fit to the data since for their comparison ${\hat{\rho }}_{\mathrm{RF}}=0.96$ which is very close to 1. This supports the conclusion based on the correctly classified responses that the LLHM and LCL models have similar fit. However, it is in contrast to the statistical significance of the likelihood-ratio statistic comparing these same two models which suggests that the large sample size may be mainly responsible for the significance of the likelihood-ratio test.

## 4 Addictiveness of tobacco products study

### 4.1 Data

The data comes from the Population Assessment of Tobacco and Health, a national longitudinal survey conducted in the United States (Hyland et al., 2017). We focus on the adolescents who had used at least one tobacco product prior to study entry. Their knowledge about the addictiveness of different tobacco products was assessed at Wave 1 of the survey. Our sample consists of 1,641 participants who were asked to rate the likelihood of someone becoming addicted to each of three less commonly used tobacco products (cigars, hookah, smokeless tobacco) on a five-point ordinal scale (1 = very unlikely, 2 = somewhat unlikely, 3 = neither likely nor unlikely, 4 = somewhat likely, 5 = very likely) with the option to choose additional DK category.

Figure 3 shows the three selected dependent variables. Table 5 presents descriptive statistics of the sample. Age of the respondents was categorized as 12–14 or 15–17 years old. About two thirds of the sample was in the older age group and there were slightly more males than females (54% vs. 46%). Close to 20% were current users of cigarettes. There is marked tendency to provide ordinal ratings in the higher categories (towards perception of greater addictiveness). The proportion of DK responses was relatively high (between 21% and 49%) and notably higher than in the Fintech knowledge case study.

**Figure 3.**
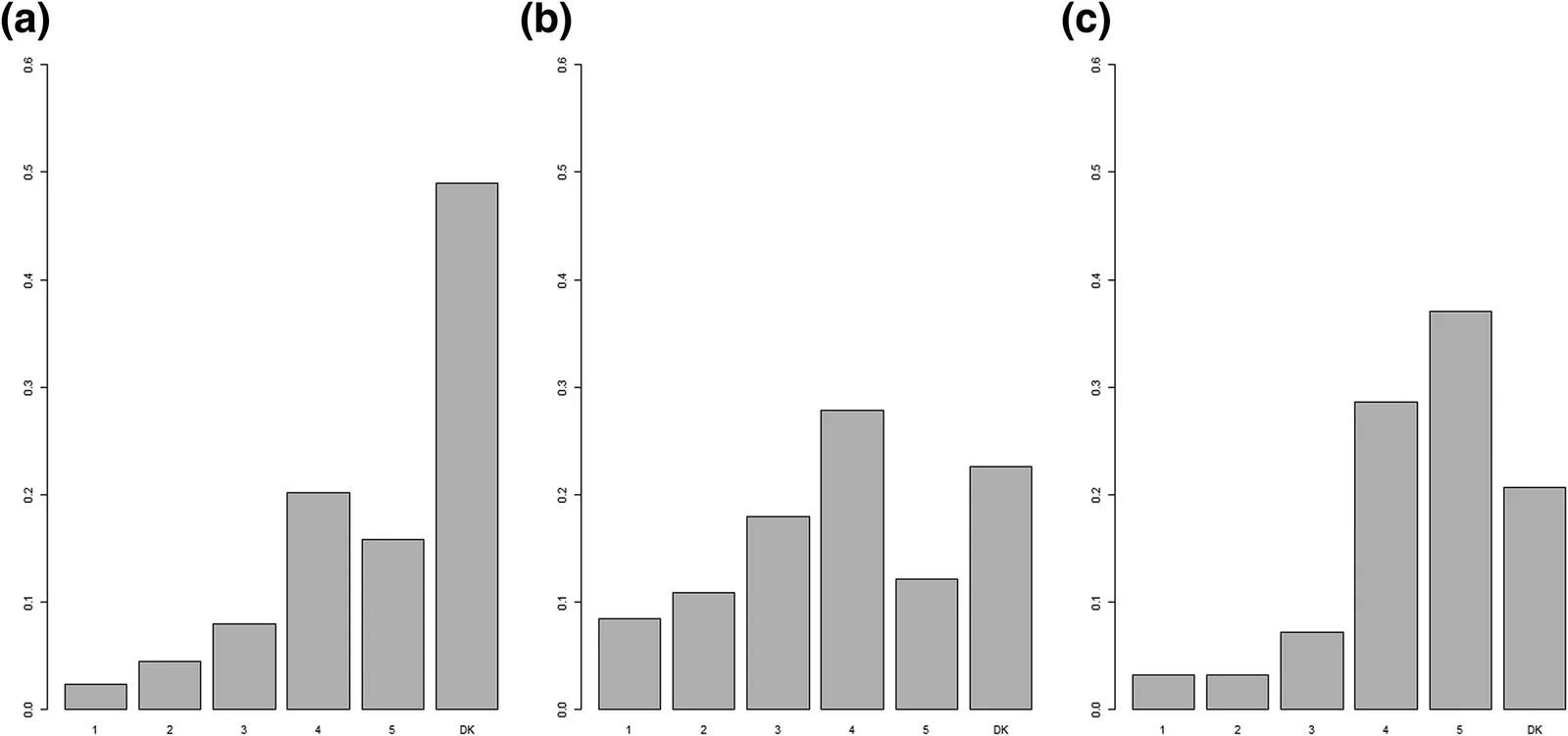
Frequency distribution of the addictiveness of *cigars* (a), *hookah* (b), *smokeless tobacco* (c) variables in the tobacco products study.

**Table 5.** Main descriptive statistics of the addictiveness of tobacco products data

		*n*	%
Gender			
	Female	755	46.0
	Male	886	54.0
Age			
	12–14 years old	619	37.7
	15–17 years old	1,022	62.3
Current user of			
cigarettes			
	Yes	310	18.9
	No	1,331	81.1

### 4.2 Results

Preliminary analyses indicated that current use of cigarettes was related to likelihood of responses in the DK category and of the location of the ordinal responses about the addictiveness of the less frequently used tobacco products. Age group appeared to be related to between-subject heterogeneity and the tendency to choose more extreme categories. Thus, in our multivariate analyses, we applied all three model formulations to the responses regarding the addictiveness of cigars, hookah and smokeless tobacco. We employed adaptive Gaussian quadrature with 10 quadrature points per dimension to ensure accurate estimation. In our analyses, we included current cigarette use (yes vs. no) as a regression predictor for DK responses and for ordinal responses, while age was incorporated as a predictor of the shift and scale components in Formulations 2 and 3.

The best-fitting model according to BIC was the logit cumulative-logit model (LCL, Formulation 1) with BIC=13,103 which is smaller than the corresponding measures for the location-scale (LLCM, Formulation 2) and the location-shift models (LLHM, Formulation 3): BIC=13,123 and BIC=13,116, respectively. Likelihood-ratio tests comparing the nested models confirm this conclusion. Thus, for these data, there is no indication of substantial residual between-subject heterogeneity in responses and differences in response styles. Table 6 presents the estimated parameters along with their standard errors for the best-fitting model (i.e. the LCL model, Formulation 1, in the last column) and in the corresponding cumulative-logit model that ignores the DK option. The models indicate that current cigarette smokers are more likely to perceive alternative tobacco products as less addictive. The effect is statistically significant at 0.05 significance level for hookah ($\beta_{21}=0.38$, SE=0.16, p-value=0.02) and a trend for cigars ($\beta_{11}=0.48$, SE=0.27, p-value=0.08). Current cigarette smokers exhibit a higher tendency to choose DK responses relative to non-smokers, which may suggest either limited knowledge of alternative tobacco products or, perhaps more likely, a deliberate avoidance of responding.

**Table 6.** Results from addictiveness of tobacco products data example

Component	Parameter	Joint Cumulative Logit	Joint Cumulative Logit and
		Ignoring Don’t Know	Don’t Know (LCL model)
		Est(SE)	Est(SE)
Rating vs. Don’t know			
	Intercept ($\beta_{00}$)	–	0.97 (0.04)
	Current smoker ($\beta_{01}$)	–	− 0.44 (0.09)
	Random intercept loading ($\lambda_{0}$)	–	0.61 (0.08)
	Correlation of random intercepts (*ρ*)	–	− 0.25 (0.11)
Cigars			
	Threshold 1 ($\tau_{11}$)	− 4.35 (0.37)	− 3.95 (0.29)
	Threshold 2 ($\tau_{12}$)	− 2.89 (0.26)	− 2.52 (0.20)
	Threshold 3 ($\tau_{13}$)	− 1.46 (0.17)	− 1.21 (0.13)
	Threshold 4 ($\tau_{14}$)	1.25 (0.16)	1.23 (0.14)
	Current use of cigarettes ($\beta_{11}$)	0.52 (0.37)	0.48 (0.27)
	Random intercept loading ($\lambda_{1}$)	1.67 (0.25)	1.59 (0.22)
Hookah			
	Threshold 1 ($\tau_{21}$)	− 2.99 (0.22)	− 2.68 (0.15)
	Threshold 2 ($\tau_{22}$)	− 1.77 (0.16)	− 1.49 (0.11)
	Threshold 3 ($\tau_{23}$)	− 0.47 (0.12)	− 0.18 (0.08)
	Threshold 4 ($\tau_{24}$)	1.95 (0.17)	2.08 (0.14)
	Current use of cigarettes ($\beta_{21}$)	0.26 (0.32)	0.38 (0.16)
	Random intercept loading ($\lambda_{2}$)	1.35 (0.18)	1.26 (0.15)
Smokeless tobacco			
	Threshold 1 ($\tau_{31}$)	− 4.04 (0.30)	− 3.80 (0.22)
	Threshold 2 ($\tau_{32}$)	− 3.26 (0.24)	− 2.97 (0.18)
	Threshold 3 ($\tau_{33}$)	− 2.16 (0.18)	− 1.96 (0.13)
	Threshold 4 ($\tau_{34}$)	0.05 (0.11)	0.21 (0.08)
	Current user of cigarettes ($\beta_{31}$)	0.12 (0.33)	0.06 (0.17)
	Random intercept loading ($\lambda_{3}$)	1.24 (0.19)	1.29 (0.17)

There is moderate heterogeneity between individuals in their responses, as indicated by the factor loading values for the random intercepts that hover around 1. The positive signs of the factor loadings for the random intercepts in the ordinal responses suggest a tendency for individuals who provide higher ratings on one question to also give higher ratings on other questions, and similarly, lower ratings tend to be consistent across responses. There is also a moderate correlation between providing a rating on a question and how high the rating is on other questions, as indicated by the small but significant correlation of the two random intercepts (ρ=−0.25, SE=0.11, p-value=0.02). A tendency to select DK on one question is associated with slightly elevated ratings on other items, suggesting a potential pattern in response behaviour characterized by a tendency to pick higher categories in the presence of uncertainty.

Neglecting the DK options appears to significantly alter the estimates of fixed effects compared to the full multivariate model that includes DK responses. Notably, the current status of the cigarette user is not significantly associated with any of the ordinal responses in the model that ignore DK. This suggests that when there is sizeable proportion of DK responses, ignoring those responses may lead to bias in the estimates. Standard error estimates are also noticeably larger in the model ignoring DK compared to the joint model. This potential bias and efficiency loss is further examined in the simulation study presented in the next section of the manuscript.

## 5 Simulation study

### 5.1 Methods

We used a simulation study to evaluate the performance of the estimation algorithms for the proposed formulations of the location-scale and location-shift models and to assess potential bias and efficiency loss from ignoring the DK options. We considered six scenarios:

**Scenario 1**: We simulated data following the joint location-shift model with DK (LLHM, Formulation 3) with 1,000 individuals. We used the same covariates for the different parts of the model as in the Fintech knowledge case study. Values for all parameters were also based on the case study. Gender was coded as 1 or 0 and split evenly and randomly between the observations in the data set. Age was generated according to a normal distribution centred at 27 with a standard deviation of 3. 20% of the observations were coded as having a university degree in economics and the remaining 80% did not. 10%, 15%, 25%, and 50% of the observations were coded at each level of the family component predictor respectively to approximate the actual distribution in the case study. Of note, the proportion of DK responses in this setting is about 15%. 200 data sets were generated according to this scenario and four models were fit to each generated data set using adaptive Gaussian quadrature in SAS PROC NLMIXED:

- the true LLHM model (Formulation 3),
- the corresponding location-shift model ignoring the DK option,
- the cumulative-logit model with DK option (LCL model, Formulation 1),
- the corresponding cumulative-logit model ignoring the DK option.

We calculated bias, relative bias, mean squared error (MSE) and coverage probabilities of 95% confidence intervals for the two location-shift models (with and without DK option). We also performed likelihood ratio tests comparing the location-shift model (LLHM, Formulation 3) to the corresponding simpler cumulative-logit model (LCL, Formulation 1) and also calculated the geometric mean index of relative model fit of those two models.

**Scenario 2**: We simulated data following the location-scale model with DK (LLCM, Formulation 2) with 1,000 individuals. As in Scenario 1, we used the same covariates for the different parts of the model as in the case study, values based on the case study and generated the covariates according to the same process. Two hundred data sets were generated according to this scenario and four models were fit to each generated data set using adaptive Gaussian quadrature in SAS PROC NLMIXED:

- the location-scale model with DK option (LLCM, Formulation 2),
- the location-scale model ignoring the DK option,
- the cumulative-logit model with DK option (LCL, Formulation 1),
- the cumulative-logit model ignoring the DK option.

We calculated bias, relative bias, mean squared error (MSE), and coverage probabilities of 95% confidence intervals for the two location-shift models (with and without DK option). We also performed likelihood-ratio tests comparing the location-scale model (LLCM, Formulation 2) to the corresponding simpler cumulative-logit model (LCL, Formulation 1) and also calculated the geometric mean index of relative model fit between those two models.

**Scenario 3**: Same as Scenario 1 but with a total sample size of 300 individuals.

**Scenario 4**: Same as Scenario 2 but with a total sample size of 300 individuals.

**Scenario 5**: Same as Scenario 1 but with a higher percentage of DK responses (approximately 50%) to match the highest rate of DK responses in the addictiveness of tobacco products case study. We anticipate larger impact of ignoring DK options as the proportion of DK responses increases.

**Scenario 6**: Same as Scenario 3 but with a higher percentage of DK responses (approximately 50%).

### 5.2 Results

Figure 4 shows box plots of relative bias for all parameters in Scenario 1 based on the location-shift model with DK (LLHM, Formulation 3) Figure 4(A) and on the location-shift model ignoring DK Figure 4(B). Similarly, Figure 5 shows box plots of relative bias for all parameters in Scenario 5 based on the location-shift model with higher proportion of DK responses (LLHM, Formulation 3) (Figure 5A) and on the corresponding location-shift model ignoring DK (Figure 5B). Figure 6 shows coverage probabilities for all parameters in the two location-shift models with less than 20% DK responses (Figure 6A, Scenario 1) and in the two location-shift models with about 50% DK responses (Figure 6B, Scenario 5). Solid lines correspond to the models accounting for DK whereas dashed lines correspond to the models ignoring DK responses. Figures 4A and 5A demonstrate little to no bias for all parameters if the correct data-generating model is fitted to the data. The differing levels of variability are due to different types of predictors and other parameters and to the inherent variability in the data. In contrast, Figures 4B and 5B demonstrate substantial bias for the threshold and for the variance-component parameters if DK options are ignored. Notably, Figure 5 shows some bias in the regression parameter estimates for the ordinal outcomes, along with substantially greater variability across all estimates. This highlights the impact of a higher proportion of DK responses on both the bias and variance of the parameter estimates. In contrast, when percentage of DK responses is lower, regression parameters are well estimated whether or not the DK option is ignored (Figure 4).

**Figure 4.**
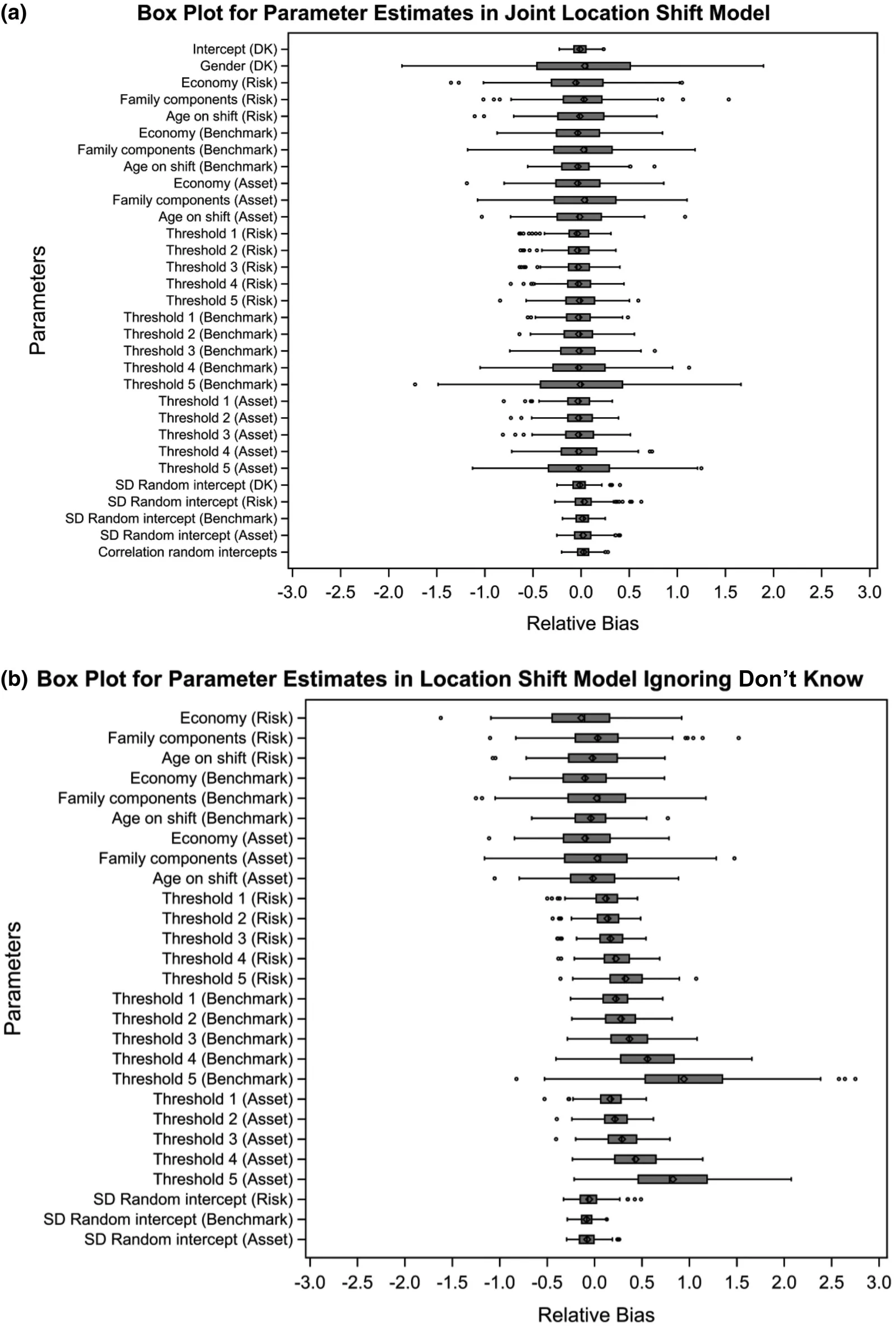
Box plots of the parameter estimates in scenario 1 of the simulation study for the location shift models. Scenario 1 corresponds to less than 20% don’t know responses. (a) Models accounting for don’t know answers. (b) Models ignoring don’t know answers.

**Figure 5.**
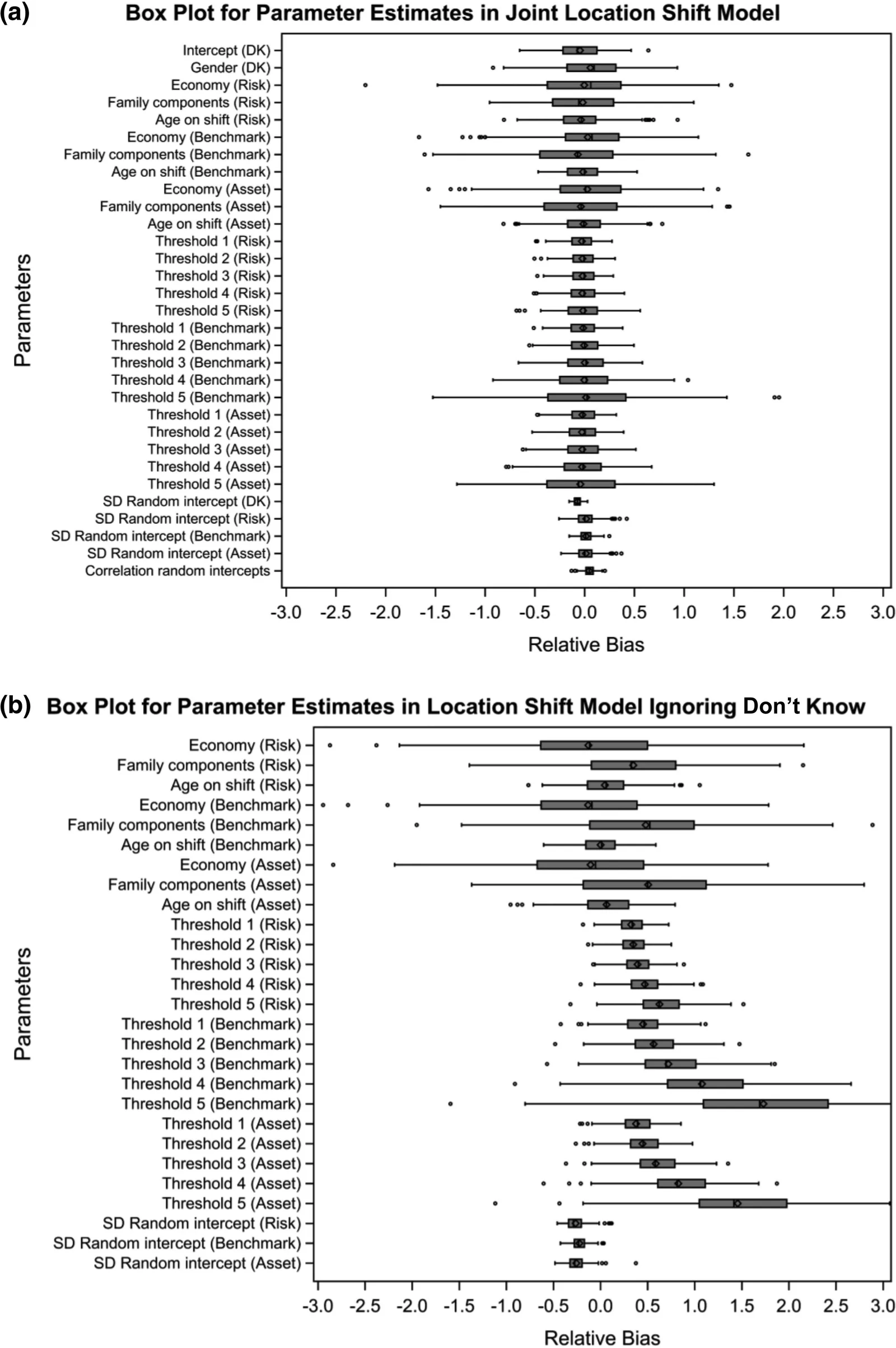
Box plots of the parameter estimates in scenario 5 of the simulation study for the location shift models. Scenario 5 corresponds to approximately 50% don’t know responses. (a) Models accounting for don’t know answers. (b) Models ignoring don’t know answers.

**Figure 6.**
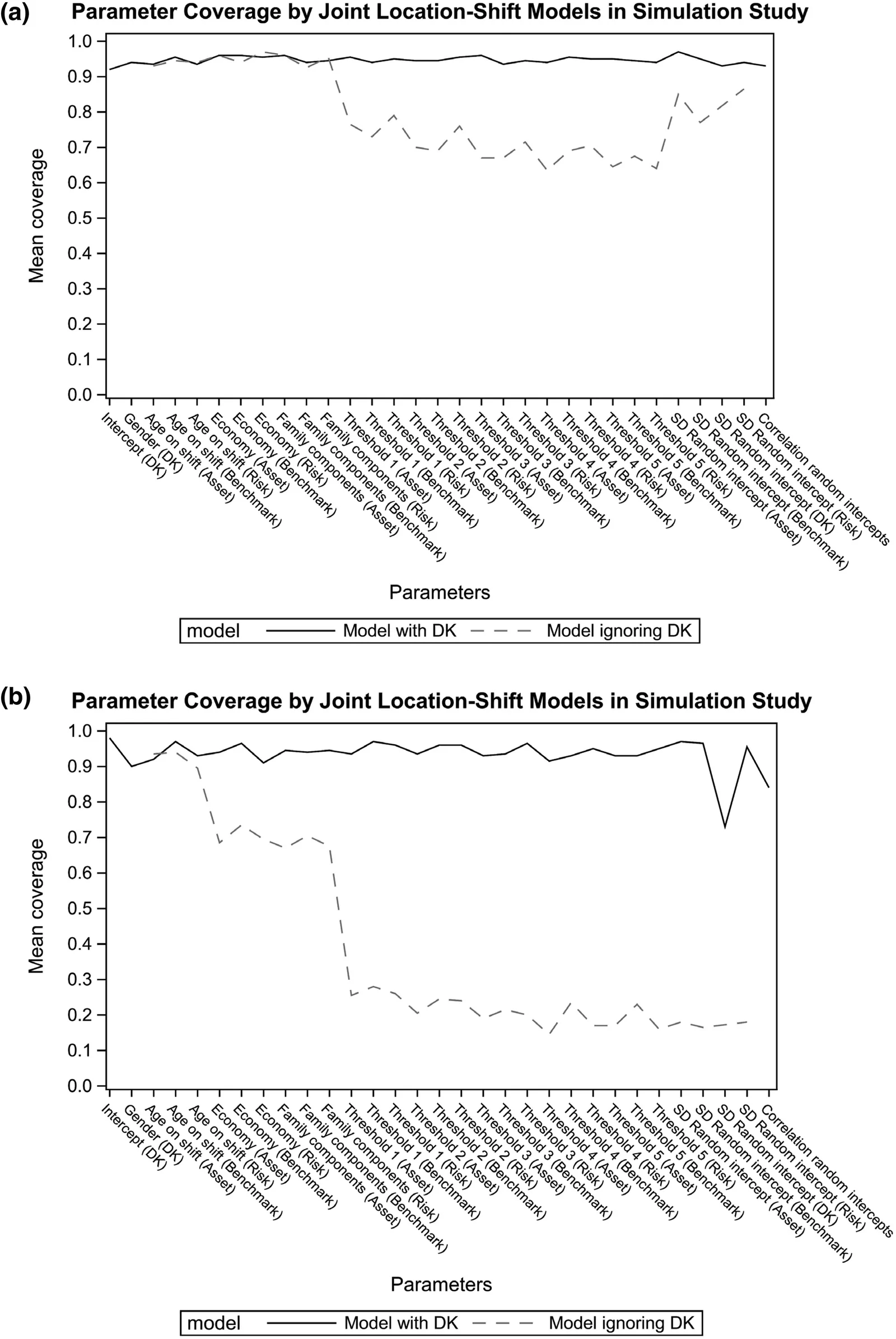
Coverage probabilities of parameter estimates in the simulation study of location shift models. (a) Less than 20% don’t know responses. (b) Approximately 50% don’t know responses.

Figure 6 also suggests that the estimation of regression parameters is relatively unaffected if DK options are ignored when the percentage of DK responses is not high (Figure 6A). However, coverage probabilities are very poor for the threshold and variance component estimates fall as far down as 40%. The problem is especially evident for the threshold parameters where all coverage probabilities are below 80%. The effect becomes even more pronounced when the proportion of DK responses approaches 50%, as illustrated in the Figure 6B. In such cases, not only are the threshold parameters poorly estimated, but the coverage probabilities of the regression coefficients for the location component of the model also fall substantially below nominal levels (around 70% rather than 95%).

In both simulation scenarios, likelihood-ratio tests are able to pick the correct model (location-shift) over the simpler cumulative-logit model 100% of the time at 0.05 significance level. However, the geometric mean index of relative model fit indicates similar fit of the two models to the cumulative-logit model (mean value of the index 0.985 and 0.981, respectively).

The results from Scenario 2 (not shown) where the location-scale model is the data-generating model are very similar to the results from Scenario 1. The results from Scenarios 3, 4, and 6 (not displayed), each involving a sample size of 300, yield results that are consistent with the patterns observed in the main analyses with larger sample size of 1,000 but bias in the estimates is more pronounced and variability is larger. The likelihood ratio tests still identify the correct model well (100% of the time in Scenario 2, 99% in Scenario 3, and 88% in Scenario 4) and the geometric mean indices of relative model fit remain high (over 90%). Results are available upon request.

## 6 Concluding remarks

We have proposed methods for handling multiple ordinal responses with DK options. These models account for individual heterogeneity and differences in response styles, as well as correlations between responses to different questions within the same individual. Covariates are incorporated both in the location and scale/shift components for the ordinal responses, as well as in the linear predictor for the DK option. Model fit indices are provided to compare and select the best-fitting model, and standard software can be employed for estimation and inference.

These methods were motivated by research questions concerning the proper management of the DK option, aiming to prevent potential biases in estimates that could arise from treating DK responses as missing data. A case study on financial knowledge and a case study of perceptions about the addictiveness of alternative tobacco products were used for illustration, and a simulation set confirmed the ability to estimate the parameters in an unbiased and efficient way.

In the financial knowledge case study, we determined that the location-shift version of the proposed models provided the best fit to the data. This model allowed us to conclude that females were more likely to respond with DK to questions about financial knowledge, individuals with economics degrees were more likely to request financial information, and that there was an increased tendency for responses in extreme categories for more mature individuals. Compared to separate analyses of the response variables, the multivariate analysis demonstrated that responses on different questions within the same individual were highly correlated and that the DK option was chosen most often by individuals who provided low ratings on other responses. Also, an individual who answered DK to one question was much more likely to answer DK to another question. Thus, the model delivered comprehensive insights into both the fixed effects and the underlying correlation structure within the data.

In the study on the perceived addictiveness of alternative tobacco products, the simplest cumulative logit model was found to offer the best fit to the data. Current smoking status emerged as a key predictor, significantly influencing both the likelihood of selecting the DK option and responses on the ordinal scale. Owing to the higher proportion of DK responses in the sample—and despite the lower correlation of the random intercepts compared to the first case study—ignoring the DK option had a more pronounced impact on the estimates, potentially introducing bias and reducing statistical power.

The simulation study we performed demonstrates that ignoring the DK options leads to substantial bias in parameter estimates. In particular, when DK answers are ignored, the estimation of threshold, intercept, and variance components becomes unreliable in all scenarios. This is intuitively expected, as the subset of individuals who provide ratings for all questions is unlikely to accurately represent the broader population of interest. As the proportion of DK responses increases, bias increases and also affects the regression parameter estimates. However, it is reassuring that when the proportion of DK responses is small, there is no noticeable bias in the estimates of the regression parameters. This result is consistent with previous literature on mixture models for ordinal data with DK responses (Gueorguieva et al., 2020) and more largely with the literature on joint modelling of different responses. It is worth noting that our simulation study is based on data generated from known models. An alternative approach is to perform an empirical simulation where an example dataset is treated as the known population and model performance is evaluated and compared for repeated simple random samples from that population. This type of simulation would demonstrate the robustness and viability of the method for at least one dataset but for relatively large data sets may be more computationally intensive.

The proposed models can be extended to longitudinal data with repeated responses on the same dependent variable. We could use fixed and random effects to model changes over time. In this situation, we could consider different random effects for each dependent variable although this increases the computational complexity of the model and would necessitate the use of alternative estimation techniques (e.g. Bayesian approach or stochastic approximation algorithms). Also, it is possible that with more complex models identifiability issues may arise.

There are alternative ways in which we could specify models for the considered scenarios. We may consider different link functions for a more flexible modelling of both polytomous and binary responses. We could also focus on discrete rather than normal distribution for the random effects (Skrondal & Rabe-Hesketh, 2004), mixtures of normal distributions (Caffo et al., 2007; Zhang et al., 2008), or log-gamma distribution appropriate for negatively skewed data (Tchetgen & Coull, 2006). However, it is possible that the effects of mis-specification of random effects distributions on inferences for effects other than the intercept are small (Agresti et al., 2004). In our data examples and simulation study, we observed that different models provided robust results regarding inference for regression parameters when proportion of DK responses is small in contrast to inference for thresholds and variance components which are more sensitive to the model specification.

There are also alternative models for ordinal data that could be extended to handle additional DK responses. Adjacent-categories logit and mean score models (Agresti, 2013) could be directly extended, whereas alternative probability distribution and mixture models such as the ones proposed by Biernacki and Jacques (2016) and McParland and Gormley (2016) could also be considered.

Future research could focus on developing more sophisticated variable selection methods within the context of the multivariate model, as well as adapting residual diagnostics and model fit measures to effectively handle the combination of ordinal and DK response options. In our current data applications, we employed a stagewise model-building approach, where covariates were identified separately for each outcome and for comparisons between DK and ordinal ratings. A more streamlined and efficient method may involve simultaneously selecting fixed effects while modelling the multivariate correlation structure of the data.

For model fit evaluation, one promising avenue could be the use of Pearson full-conditional residuals, which compare each observed value with its expected value, providing a more nuanced assessment of model performance. Measures that account for the ordinal nature of the responses could enhance the evaluation of model fit and predictive performance. For instance, the concordance index, various pseudo $R^{2}$ metrics, and measures based on underlying latent variables for the ordinal responses (as discussed in Agresti & Tarantola, 2018) could be adapted to incorporate the DK option. This could be achieved by employing a weighted approach that appropriately integrates the DK responses into both the ordinal submodel and the binary submodel, ensuring a more comprehensive and accurate assessment of model performance.

In conclusion, the models proposed herein provide flexible extensions to existing methods for ordinal data that enable the incorporation of DK options, between-subject variability and response styles. Model fitting is straightforward to implement with existing software and simulation studies demonstrate good statistical properties of the maximum likelihood estimation procedure.

## Data Availability

Data are not publicly available due to funder restrictions but are available from the authors upon reasonable request with permission from the relevant institution.

## Footnotes

1 Sources: www.cost.eu/actions/CA19130; www.fin-ai.eu.
